# SPRY4 acts as an indicator of osteoarthritis severity and regulates chondrocyte hypertrophy and ECM protease expression

**DOI:** 10.1038/s41536-021-00165-9

**Published:** 2021-09-17

**Authors:** Sunghyun Park, Yoshie Arai, Alvin Bello, Hansoo Park, Dohyun Kim, Kyung-Soon Park, Soo-Hong Lee

**Affiliations:** 1 Department Medical Biotechnology, Dongguk University Biomedi Campus, Goyang-si, Gyeonggi-do Republic of Korea; 2 Department of Biomedical Science, CHA University, Seongnam-si, Gyeonggi-do Republic of Korea; 3 School of Integrative Engineering, Chung-ang University, Seoul, Republic of Korea

**Keywords:** Senescence, Osteoarthritis, Cell signalling

## Abstract

Osteoarthritis (OA) causes serious changes in the metabolic and signaling pathways of chondrocytes, including the mitogen-activated protein kinase (MAPK) pathway. However, the role of sprouty RTK signaling antagonist 4 (SPRY4), an inhibitor of MAPK, in the human cartilage tissues and chondrocytes remains to be understood. Here, using SPRY4 gene delivery into healthy and degenerated chondrocytes, we elucidated the role of *SPRY4* in preventing chondrocyte hypertrophy. In addition to using the human cartilage tissues with the destabilization of the medial meniscus (DMM) model in Sprague-Dawley (SD) rats, the role of SPRY4 in cartilage tissues and chondrocytes was explored through their molecular and histological analyses. In order to determine the effects of SPRY4 on healthy human chondrocyte hypertrophy, small interfering RNA (siRNA) was used to knock down *SPRY4*. Lentiviral transduction of *SPRY4* into degenerated human chondrocytes allowed us to investigate its ability to prevent hypertrophy. *SPRY4* expression levels were higher in healthy human cartilage tissue and chondrocytes than in degenerated human cartilage tissues and hypertrophy-induced chondrocytes. The knockdown of *SPRY4* in healthy chondrocytes caused an increase in hypertrophy, senescence, reactive oxygen species (ROS) production, and extracellular matrix (ECM) protease expression. However, all these factors decreased upon overexpression of *SPRY4* in degenerated chondrocytes via regulation of the MAPK signaling pathway. We conclude that *SPRY4* is a crucial indicator of osteoarthritis (OA) severity and could play an important role in preventing OA in the cartilage by inhibiting chondrocyte hypertrophy.

## Introduction

The molecular pathogenesis of OA involves numerous molecular, cellular, and niche alterations, including chondrocyte hypertrophy, reactive oxygen species (ROS) generation, chondrocytes senescence, decrease in cell proliferation, protease activation, extracellular matrix (ECM) collapse of cartilage, inflammation, and osteophyte formation^1,2^. Of these, chondrocyte hypertrophy is a representative pathogenic symptom characterized by volume enlargement and protease activation and typically associated with early stages of OA^3,4^. Although chondrocyte hypertrophy is essential for endochondral ossification in healthy tissue, it is also implicated in the pathogenesis of OA^4^. Chondrocyte hypertrophy is tightly regulated by genes such as Indian hedgehog (*IHH*), collagen type X (*COL10*), matrix metalloproteinase-13 (*MMP13*), and runt-related transcription factor-2 (*RUNX2*)^4,5^. *RUNX2*, a member of the runt family of transcription factors that plays a crucial role in osteoblast and chondrocyte differentiation, is also tightly associated with OA development and progression^6,7^. In OA, *RUNX2* regulates chondrocyte hypertrophy by inducing ECM mineralization and vascular invasion, thereby inducing chondrocyte apoptosis, osteogenic lineage differentiation, cartilage fibrosis, and ECM protease activation^8–10^. ECM protease activation is a crucial factor in accelerating OA and is typically responsible for degrading and decomposing the cartilage’s fibrous ECM proteins such as collagen and aggrecan^3^. Many proteases such as MMP13 and disintegrin and metalloproteinase with thrombospondin motifs 5 (ADAMTS5) have been associated with OA^11,12^. Increases in *RUNX2* expression and ECM protease activation are critical biological markers of chondrocyte hypertrophy, OA progression, and cartilage collapse.

OA pathogenesis also involves the activation of specific signaling pathways that initiate a protein chain reaction. Among these, the mitogen-activated protein kinase/extracellular signal-regulated kinase (MAPK/ERK) signaling pathway is crucially responsible for OA progression^13,14^. MAPK/ERK activation and phosphorylation induce an increase in *RUNX2* expression, *MMP13* expression, chondrocyte apoptosis, and cartilage decomposition^15,16^. Therefore, the involvement of the MAPK/ERK pathway needs to be investigated further to better understand chondrocyte hypertrophy and OA.

The SPRY family comprising four known isoforms (SPRY 1, 2, 3, and 4)function as antagonists of MAPK signaling. Although the roles of *SPRY 1*, *2*, and *3* in MAPK signaling have been investigated in other studies^17,18^, the sprouty RTK signaling antagonist 4 (*SPRY4*) requires further investigation to understand its role and mechanism in various organs. Previous research has elucidated that *SPRY4*, which acts as an antagonist of MAPK signaling by inhibiting the formation of active GTP-Ras in the MAPK/ERK pathway, regulates cytokine secretion, cell proliferation, and cell differentiation^19–21^. Furthermore, we have reported that the suppression of *SPRY4* promotes osteogenic differentiation of mesenchymal stem cells by upregulating ERK 1/2 phosphorylation^22^. We have also reported that *SPRY4* knockdown induces cellular hypertrophy in mesenchymal stem cells (MSC). Both, osteogenic differentiation and cellular hypertrophy are essential processes in endochondral ossification^23,24^. Cellular hypertrophy was induced by *SPRY4*, an antagonist of MAPK/ERK signaling, in MSCs; therefore, we hypothesized that it might also have an effect on chondrocyte hypertrophy, which is crucially involved in the MAPK/ERK signaling in OA. Accordingly, we investigated the role of *SPRY4* in chondrocytes by comparing its molecular expression in healthy and degenerated cartilage obtained from human cartilage tissues and in-vivo DMM OA-induced models. Moreover, we utilized siRNA-mediated knockdown and lentiviral transduction of *SPRY4* in chondrocytes to investigate its role in hypertrophy and OA.

## Results

### *SPRY4* expression in healthy and degenerated cartilage

**Fig. 1:**
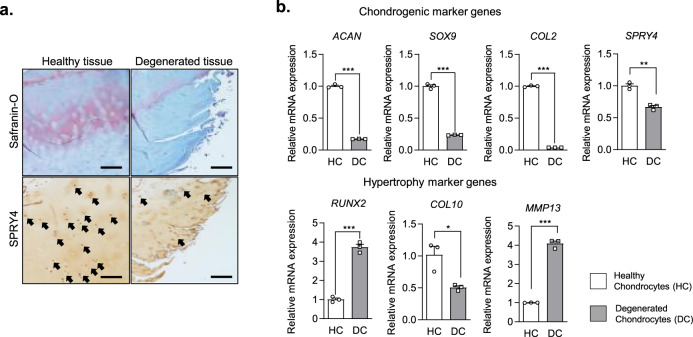
Expression of *SPRY4* in healthy and degenerated human cartilage. **a** Safranin-O staining (upper panel) and SPRY4 IHC staining (lower panel) in healthy and degenerated human cartilage. Scale bar, 100 μm. **b** Chondrogenic marker gene mRNA expression levels (top), hypertrophic marker gene mRNA expression levels (middle), and *SPRY4* mRNA expression levels (bottom) in Healthy chondrocytes (HCs) and Degenerated chondrocytes (DCs). The data represent the mean ± SEM (*n* = 3; ACAN, SOX9, COL2, and MMP13: unpaired *t*-test; RUNX2 and COL10: unpaired *t*-test with Welch’s correction; MMP13: Mann Whitney test).

In order to investigate the relationship between the *SPRY4* expression and OA, we first compared the *SPRY4* expression levels in healthy and degenerated cartilage samples isolated from patients. According to the histological analysis (Fig. 1a), glycosaminoglycan (GAG) content is lower in degenerated cartilage than in healthy cartilage. Healthy chondrocytes (HCs) and degenerated chondrocytes (DCs) were isolated from healthy and degenerated tissue, respectively. As shown in Fig. 1b, expression levels of chondrogenic marker genes (*ACAN, SOX9*, and *COL2*) were lower, whereas the expression levels of hypertrophic marker genes (*RUNX2* and *MMP13*) were higher in the DCs than in the HCs. These results support those of previous studies^25,26^. Interestingly, the SPRY4 protein expression level was notably lower in degenerated cartilage than in healthy cartilage (Fig. 1a, lower panel). The same pattern was confirmed for *SPRY4* gene expression levels in HCs and DCs (Fig. 1b). These results indicate that gene and protein expression levels of SPRY4 are associated with pathological conditions in the cartilage. The same relationship between SPRY4 expression levels and OA was also observed in the DMM animal model that mimicked the osteoarthritis bio environment (Supplementary Fig. 1).

### Effect of IL-1β treatment on HCs

*SPRY4* expression level was lower in DCs and degenerated human cartilage tissue than in HCs and healthy cartilage. Therefore, we induced hypertrophy in HCs by treating them with IL-1β. Treating HCs with IL-1β decreased the expression levels of chondrogenic marker genes (*ACAN, SOX9*, and *COL2*) (Supplementary Fig. 2a), whereas the expression levels of protease marker genes (*MMP1, MMP3*, and *MMP13*) and hypertrophic marker genes (*COL10*) increased (Supplementary Fig. 2b, c). MMP1, MMP3, and MMP13 are critical proteases involved in the degradation of cartilage ECM matrix^27^. The expression level of *MMP13* in IL-1β-treated cells was increased compared to that in control cells. These results confirmed that IL-1β effectively induces chondrocyte hypertrophy. Furthermore, IL-1β treatment significantly decreased *SPRY4* gene expression compared to that in the control (Supplementary Fig. 2d, e). Therefore, we conclude that *SPRY4* plays a significant role in chondrocyte hypertrophy and the expression of inflammatory-related cytokines.

### Effect of *SPRY4* knockdown on cellular morphology, hypertrophy, and senescence in HCs

**Fig. 2:**
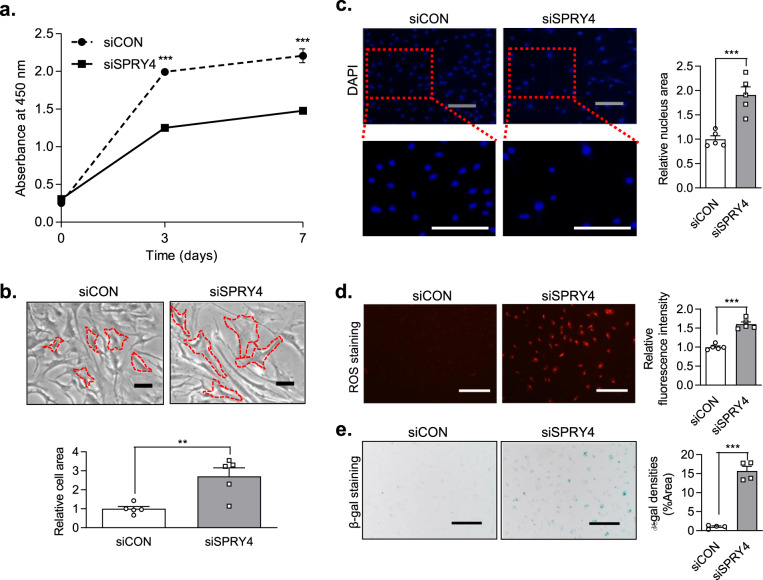
Effect of *SPRY4* suppression on chondrocyte hypertrophy in HCs. **a** CCK-8 cell proliferation assay of siCON-treated and siSPRY4-treated HCs by time (*n* = 3, unpaired *t*-test with Welch’s correction). **b** Microscopic images of siCON-treated and siSPRY4-treated HCs (*n* = 5, unpaired *t*-test) Scale bars, 100 μm. **c** DAPI staining of siCON-treated and siSPRY4-treated HCs (*n* = 5, unpaired t-test with Welch’s correction) Scale bars, 100 μm. **d** ROS staining of siCON-treated and siSPRY4-treated HCs (*n* = 5, unpaired *t*-test with Welch’s correction) Scale bars, 250 μm. **e** β-gal staining of siCON-treated and siSPRY4-treated HCs (*n* = 4, unpaired *t*-test) Scale bars, 250 μm. All data represent the mean ± SEM. Abbreviations: CCK-8, cell counting kit-8; DAPI, 4′,6-diamidino-2-phenylindole; ROS, reactive oxygen species; β-gal, β-galactosidase.

In order to confirm the effect of *SPRY4* on chondrocytes, we investigated changes in cellular morphology, hypertrophy, and senescence in the HCs after a siRNA-mediated knockdown of *SPRY4*. siRNA-SPRY4 (siSPRY4) treatment significantly decreased *SPRY4* gene expression by up to 30% (Supplementary Fig. 3). The growth rate of siSPRY4-treated HCs over 7 days was 33.2% lower than that of cells treated with control siRNA (siCON) (Fig. 2a). Using microscopic imaging and DAPI staining, we determined that siSPRY4 treatment altered the cellular morphology of HCs. The ImageJ software was used to measure the size of the cell that was significantly increased by approximately 3 times and the size of the cell nucleus was doubled compared to that of the control (Fig. 2b, c). Moreover, the expression level of reactive oxygen species (ROS) and senescence-associated β-gal staining were higher in siSPRY4-treated HCs than those in the control; these results were confirmed quantitatively (Fig. 2d, e). These results demonstrate that knockdown of *SPRY4* increases hypertrophy-associated factors, such as cellular morphology, ROS generation, and senescence.

### Effect of *SPRY4* knockdown on chondrogenic and hypertrophic marker gene expression levels in HCs

**Fig. 3:**
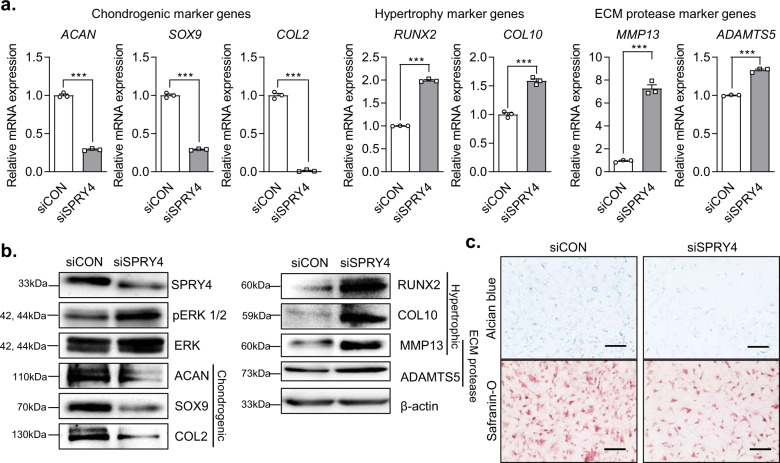
Effect of *SPRY4* suppression on the OA-related biological properties of HCs. **a** mRNA expression analysis of various chondrogenic, hypertrophic, and ECM protease markers in siCON-treated and siSPRY4-treated HCs. The data represent the mean ± SEM (*n* = 3, unpaired *t*-test with Welch’s correction). **b** Western blot protein analyses of various chondrogenic, hypertrophic, and ECM protease markers in siCON-treated and siSPRY4-treated HCs. **c** Alcian blue and Safranin-O staining of siCON-treated and siSPRY4-treated HCs. Scare bar, 100 μm.

We also investigated mRNA and protein expression levels of chondrogenic, hypertrophic, and ECM protease markers, as well as the GAG content, in the HCs. The trends observed for mRNA expression were similar to those for protein expression. *SPRY4* knockdown decreased the expression levels of chondrogenic marker genes (*ACAN*, *SOX9*, and *COL2*) and increased the expression levels of hypertrophic marker genes (*RUNX2* and *COL10*) and ECM protease marker genes (*MMP13* and *ADAMTS5*) compared to those of the control (Fig. 3a). Protein analysis showed that chondrogenic marker protein levels (ACAN, SOX9, and COL2) were decreased, whereas hypertrophic marker protein levels (RUNX2, COL10, and MMP13) and ECM protease marker protein levels (MMP13 and ADAMTS5) were increased in siSPRY4-treated HCs (Fig. 3b). Interestingly, pERK 1/2 protein expression, which is related to RUNX2 and MMP expression^16,28^, was increased in siSPRY4-treated HCs. In contrast, other MAPK signaling proteins such as p38 and JNK, as well as their phosphorylated forms showed no significant differences between the control group and the siSPRY4-treated HCs (Supplementary Fig. 4). These results indicate that SPRY4 regulates chondrocyte hypertrophy and OA progression through the ERK-MAPK signaling pathway. Alcian blue staining and safranin-O staining revealed that GAG content decreased in siSPRY4-treated HCs compared to that in the control (Fig. 3c). These results imply that *SPRY4* knockdown affects chondrocyte hypertrophy and senescence.

### Effect of *SPRY4* overexpression on cellular morphology, hypertrophy, and senescence in DCs

**Fig. 4:**
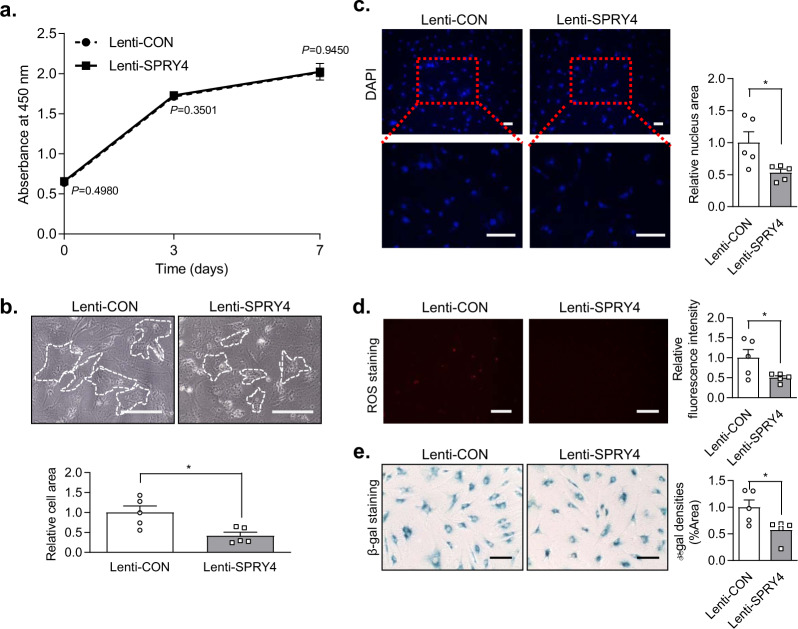
Effect of *SPRY4* overexpression on hypertrophy in DCs. **a** CCK-8 cell proliferation assay for the control lentivirus (Lenti-CON)-treated and *SPRY4* lentivirus (Lenti-*SPRY4*)-treated DCs by time (*n* = 3, unpaired *t*-test with Welch’s correction). **b** Microscopic images of Lenti-CON-treated and lenti-*SPRY4*-treated DCs (*n* = 5, unpaired *t*-test with Welch’s correction) Scale bars, 200 μm. The data represent the mean ± SEM (**c**) DAPI staining of Lenti-CON-treated and Lenti-*SPRY4*-treated DCs (*n* = 5, unpaired *t*-test with Welch’s correction) Scale bars, 100 μm. **d** ROS staining of Lenti-CON-treated and Lenti-*SPRY4*-treated DCs (*n* = 5, unpaired *t*-test) Scale bar, 200 μm. **e** β-gal staining of Lenti-CON-treated and Lenti-*SPRY4*-treated DCs (*n* = 5, Mann Whitney test) Scale bar, 200 μm. All data represent the mean ± SEM. Abbreviations: CCK-8, cell counting kit-8; DAPI, 4′,6-diamidino-2-phenylindole; ROS, reactive oxygen species; β-gal, β-galactosidase.

In order to understand the role that *SPRY4* plays in regulating hypertrophic changes and senescence in HCs and DCs, we utilized a lentiviral vector to overexpress *SPRY4* in DCs. First, the efficiency of the lentiviral delivery system was examined. Lenti-SPRY4 increased *SPRY4* mRNA expression level compared to that of the negative control (Supplementary Fig. 5a); this increase was also observed by IF staining (Supplementary Fig. 5b). Using FACS analysis, the transduction efficiency of Lenti-SPRY4 was determined to be 80.23% (Supplementary Fig. 5c). After transfecting the lentiviral vectors into the DCs, we investigated the effect of *SPRY4* overexpression on cellular morphology, hypertrophy, and senescence. *SPRY4* overexpression in DCs had a negligible effect on the cell growth rate compared to that of the control (Fig. 4a). However, as confirmed by quantification, *SPRY4* overexpression reduced the size of the cell body (Fig. 4b) and the nucleus in the DC compared to that in the control (Fig. 4c). ROS level and senescence, indicated by β-gal staining, of the DCs were also decreased by *SPRY4* overexpression (Fig. 4d, e). These results suggest that *SPRY4* overexpression might be useful in restoring chondrogenic characteristics to the DCs in terms of morphology, ROS level, and senescence.

### Effect of *SPRY4* overexpression on chondrogenic and hypertrophic marker genes expression in DCs

**Fig. 5:**
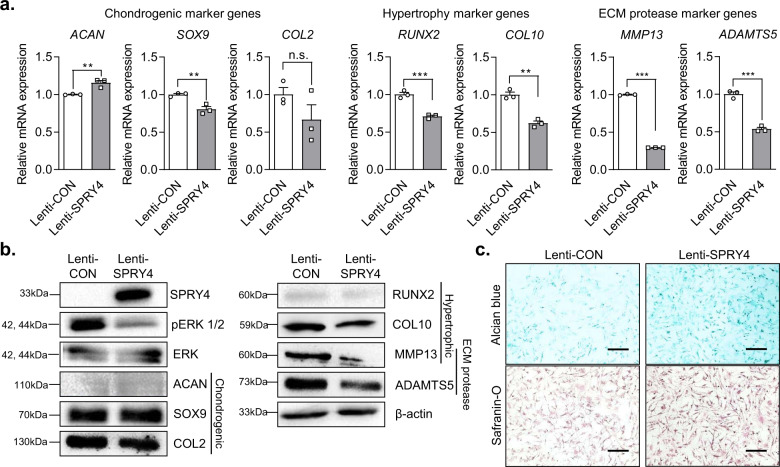
Effect of *SPRY4* overexpression on OA-related biological properties of DCs. **a** mRNA expression analysis of various chondrogenic, hypertrophic, and ECM protease markers in Lenti-CON-treated and Lenti-*SPRY4*-treated DCs. The data represent the mean ± SEM (*n* = 3; ACAN and MMP13: unpaired *t*-test; SOX9, COL2, RUNX2, COL10, and ADAMT5: unpaired *t*-test with Welch’s correction). **b** Western blot protein analyses of various chondrogenic, hypertrophic, and ECM protease markers in Lenti-CON-treated and Lenti-*SPRY4*-treated DCs. **c** Alcian blue and Safranin-O staining of Lenti-CON-treated and Lenti-*SPRY4*-treated DCs. Scale bars, 200 μm.

We determined the mRNA and protein expression levels of chondrogenic, hypertrophic, and ECM protease markers, as well as the GAG content in DCs. The expression levels of hypertrophic marker genes (*RUNX2* and *COL10*) and ECM protease marker genes (*MMP13* and *ADAMTS5*) in DCs were significantly decreased by *SPRY4* overexpression (Fig. 5a). However, *SPRY4* overexpression induced no notable differences in the expression levels of chondrogenic marker proteins (ACAN, SOX9, and COL2). In contrast, *SPRY4* overexpression distinctly decreased the expression levels of hypertrophic marker proteins (RUNX2 and COL10), ECM protease marker proteins (MMP13 and ADAMTS5), and the pERK 1/2 protein (Fig. 5b). Alcian blue and safranin-O staining revealed that *SPRY4* overexpression increased the GAG content in DCs compared to that in the control (Fig. 5c). These results demonstrate that *SPRY4* overexpression in DCs has a more notable effect on hypertrophic and ECM protease markers than on chondrogenic markers. Therefore, we concluded that overexpressing *SPRY4* might be a useful strategy to restore chondrogenic characteristics to DCs, especially in terms of hypertrophy. We confirmed that *SPRY4* affects OA progression by inhibiting chondrocyte hypertrophy and senescence.

## Discussion

During OA progression, the MAPK/ERK signaling pathway regulates chondrocyte hypertrophy through the phosphorylation of ERK 1/2, leading to increased expression of *RUNX2* and multiple MMPs^16,29^, which are crucial in regulating chondrocyte hypertrophy and inducing OA. Beier et al. reported that the MAPK signaling cascade also leads to the activation of chondrocyte hypertrophic markers, such as COL10^30^. Furthermore, MAPK/ERK signaling induces senescence-related accumulation of ROS and cell death via intrinsic or extrinsic apoptotic pathways, including mitochondrial cytochrome C release, caspase-8 activation, and autophagic vacuolization^31^. It has been established that *SPRY4* is an inhibitor and regulator of the MAPK/ERK signaling pathway^32^. Therefore, we hypothesized that *SPRY4* plays a critical role in OA by influencing chondrocyte hypertrophy and senescence.

In order to determine whether *SPRY4* is related to OA, we compared its expression levels in healthy and degenerated cartilage tissues. Interestingly, the expression level of *SPRY4* was significantly lower in degenerated cartilage tissues compared to that in the healthy cartilage tissues. This finding supported our hypothesis that the expression of *SPRY4* in the cartilage is associated with OA progression, characterized by increased chondrocyte hypertrophy and inflammatory conditions^33,34^. In order to compare the differences between the HCs and DCs further, we measured the expression levels of several chondrogenic and hypertrophic markers. As expected, chondrogenic markers (*ACAN, SOX9*, and *COL2*) were upregulated in HCs, whereas hypertrophic markers (*RUNX2* and *MMP13*) were upregulated in the DCs. Notably, contradictory results exist on the use of *ACAN* and *COL2* as chondrogenic markers, which primarily depend on the developmental phase of OA. However, both *ACAN* and *COL2* are still widely accepted as chondrogenic markers and therefore used in this study^35,36^. The expression of *COL10*, however, was found to be slightly lower in DCs, presumably because of population variation. Gene expression in samples of human cartilages is known to differ depending on the donor’s age, genetic background, and pathologic conditions^37^.

In order to determine whether the induction of hypertrophy would affect the expression levels of *SPRY4* in chondrocytes, we treated the HCs with IL-1β. As expected, IL-1β treatment in HCs caused a significant reduction in chondrogenic marker genes; however, it upregulated ECM protease expression levels and increased chondrocyte hypertrophy. Interestingly, *SPRY4* expression in IL-1β-treated HCs was also reduced. This result supports the idea that *SPRY4* expression is a crucial indicator of cartilage health and chondrocyte hypertrophy. Meanwhile, we found that *RUNX2* expression was not significantly different in untreated and IL-1β-treated HCs, presumably because of the inhibition of *RUNX2* by IL-1β. Ding J et al. reported that treating human mesenchymal stem cells (hMSCs) with IL-1β increased alkaline phosphatase activity and mineralization and inhibited *RUNX2*^38^. Although chondrocyte hypertrophy induces OA, it is also well established that it increases the secretion of alkaline phosphatase, osteocalcin, and osteopontin similar to endochondral ossification in osteogenesis in hMSCs^39^. Therefore, considering the similarities in endochondral ossification in hMSCs and hypertrophic chondrocytes, a similar result might be observed.

Next, we investigated the effect of *SPRY4* on HCs and DCs in vitro using *SPRY4* knockdown and overexpression studies. We utilized siSPRY4 to knock down *SPRY4* and lenti-*SPRY4* to induce its overexpression. Downregulation of *SPRY4* induced chondrocyte hypertrophy in HCs and increased ERK 1/2 phosphorylation. In contrast, upregulation of *SPRY4* inhibited hypertrophic changes in DCs and decreased ERK 1/2 phosphorylation. Based on the mRNA and protein expression levels of hypertrophic markers and GAG staining analysis, the upregulation of *SPRY4* in DCs significantly reduced chondrocyte hypertrophy compared to that in the control. However, chondrogenic marker gene expression levels were not significantly affected *SPRY4* upregulation. We believe that because the DCs have already undergone hypertrophy, they primarily express *RUNX2*, *COL10*, bone sialoprotein (*BSP*), and *MMP13* while expressing fewer chondrogenic factors, such as *ACAN*, *SOX9*, and *COL2*^40^. Moreover, many studies have reported that MAPK signaling induces chondrocyte hypertrophy, but not chondrocyte regeneration^41–44^. Taken together, our results suggest that *SPRY4* overexpression is critical to OA progression through regulation of the MAPK signaling pathway by inhibiting ERK1/2 phosphorylation. In our previous study, we confirmed that *SPRY4* knockdown directly affects the increase in ERK1/2 phosphorylation after u0126 treatment^22^. However, we only investigated the possible role of *SPRY4* as a therapeutic for OA. Therefore, our results about the effects of *SPRY4* overexpression on MAPK/ERK signaling to require further validation and additional drug targeting to elucidate its complete mechanism.

Furthermore, various studies involving chemicals and scaffolds have focused on MAPK signaling with chondrocytes hypertrophy. For example, Indira et al. reported that MAPK signaling was regulated by u0126 with hyaluronic acid (HA); they reported positive synergistic effects on both chondrocyte hypertrophy and a rat meniscectomy (MSX) OA-induced model^45^. Wang et al. reported that chondrocyte hypertrophy was inhibited and redifferentiated by PD0325901^46^. However, studies applying chemicals and scaffolds may not be able to solve the fundamental problem in OA. Overexpression of *SPRY4* might be a good therapeutic candidate because it prevents hypertrophy on a genetic level. We also confirmed that *SPRY4* overexpression did not have any negative effects on chondrocyte viability (Supplementary Fig. 6), which indicates its safety for long-term use. Although further studies and additional experiments are required to validate its effectiveness and efficiency, we believe that *SPRY4* will ultimately be a strong candidate for direct OA treatment.

In this study, we compared *SPRY4* expression in healthy and degenerated (OA) human cartilage and found that it was higher in healthy cartilage tissue. IL-1β treatment in HCs significantly reduced *SPRY4* expression. Furthermore, although the siRNA-mediated downregulation of *SPRY4* in HCs reduced characteristics such as GAG content, it increased hypertrophic markers such as RUNX2 and MMP13. In contrast, the upregulation of *SPRY4* in DCs decreased hypertrophy. In conclusion, we confirmed *SPRY4* as a potential indicator of OA severity based on the difference in SPRY4 expression between healthy and degenerated cartilage tissues. We also confirmed that *SPRY4* regulates chondrocyte hypertrophy, which when inhibited has a preventive effect on OA progression.

## Methods

### Human chondrocyte isolation and culture

Human cartilage was obtained by manual isolation from the knee of donor patients, who provided written informed consent to the CHA Stem Cell Institute with approval from the Institutional Review Board of the CHA University Hospital Ethics Committee (IRB NO. 2014-096). Healthy and degenerated cartilage tissues were separated based on a previously described protocol^26^. Briefly, the white-colored, intact, abundant hyaline cartilage tissues were classified as relatively healthy tissue, whereas the red-colored, collapsed, fibrous cartilage tissues were classified as relatively degenerated tissue. Cells isolated from the healthy cartilage were denoted as healthy chondrocytes (HCs), while cells from degenerated tissues were denoted as degenerated chondrocytes (DCs). Both tissues were washed three times with phosphate-buffered saline (PBS), and then chopped using a surgical blade until they become fine particles. Chopped tissues were re-washed in PBS and digested with 0.5 mg/ml collagenase (Sigma Aldrich, MO) in DMEM low-glucose media (HyClone, MA) containing 1% antibiotics (P/S) overnight (O/N) at 37 °C in a 5% CO_2_ incubator. The cells were separated from undigested tissues using filtration, washed with 1× PBS, and resuspended in DMEM low-glucose medium supplemented with 10% FBS and 1% P/S and cultured at 37 °C in a 5% CO_2_ incubator. After primary chondrocytes were attached to the culture dish, the growth medium was changed every three days. At 80% confluency, chondrocytes were harvested with 0.5% Trypsin-EDTA (Invitrogen, NY) and passaged at a seeding density of 3 × 10^4^ cells/cm^2^. Whole-cell experiments were performed at passage 3.

### Interleukin 1β (IL-1β) treatment

Human-derived chondrocytes were used for IL-1β treatment at passage 3. Chondrocytes were seeded at a density of 1 × 10^5^ per well in a 6-well plate (Falcon, NC). After O/N incubation, chondrocytes were incubated for 12 h in 0.5% FBS low-glucose DMEM. Subsequently, 200 ng of IL-1β (R&D, MN) was added to each well and the chondrocytes (*n* = 3) were incubated for an additional 2 days.

### Cytological and histological staining

Chondrocytes and cartilage samples were fixed with 4% (w/v) paraformaldehyde (PFA; Biosesang, Seongnam, Korea). In the case of cartilage, samples were decalcified in a decalcification solution (BBC Biochemical, WA) for 1 week and processed as described previously^26^. After fixation, whole chondrocytes and cartilage specimens were paraffinized and hydrated before staining. Specimens were stained with 1% alcian blue in 3% acetic acid (pH 2.5) for 1 h. Cartilage specimens were also stained with 0.1% Safranin-O for 1 h and counterstained with 0.002% fast green solution for 3 min. GAG matrices that formed in specimens were observed using light microscopy. For immunohistochemistry (IHC) and immunofluorescence (IF) staining, specimens were treated with a pepsin solution for antigen retrieval. Subsequently, samples were incubated in a blocking solution for 1 h and then incubated for 12 h in a 1:200 anti-SPRY4 primary antibody solution. The samples were then washed and treated for 1 h with a secondary antibody solution for IHC or Alexa Fluor 488 goat anti-rabbit IgG (Invitrogen, CA) for IF. In the case of IHC, antibody-antigen binding was detected using the streptavidin-HRP solution and DAP reaction solution. All IHC analyses were performed using a GBI kit (GBI Labs, WA). For IF, fluorescence signals were detected with a Cytation 3 Cell Imaging Reader (Biotek, VT). For DAPI staining, fixed chondrocytes were treated with 20 μl of 4’,6-diamidino-2-phenylindole (DAPI) solution (Invitrogen, CA) per 1 ml of PBS. After a 10-min incubation, fluorescence was detected with a Cytation 3 Cell Imaging Reader. For reactive oxygen species (ROS) staining, 5 × 10^4^ chondrocytes were seeded and cultured on 12-well plates for 1 week. After washing once with PBS, PBS containing 5 μM of Mito SOX Red (M36008, Invitrogen) probe was added and incubated for 15 min at 37 °C in the dark. After washing twice with PBS, red fluorescence was detected using a fluorescence microscope (Olympus, Tokyo, Japan). Senescence-associated β-galactosidase (β-gal) staining was performed with a staining kit (Cell Signaling Technology, MA). Briefly, chondrocytes were incubated for 7 days after seeding. Subsequently, chondrocytes were fixed and stained according to the manufacturer’s protocol. Blue-stained cells were counted as positive for senescence. Subsequently, stained specimens were mounted (StatLab, TX) and observed using light microscopy. Whole staining results and microscopic images (*n* = 5) were selected randomly, and size or fluorescence intensity was measured using the ImageJ software.

### Real-time quantitative polymerase chain reaction (RT-qPCR)

Chondrocytes were treated with 1 ml of TRIzol reagent (Invitrogen, CA) (*n* = 3). Total RNA was extracted and then reverse-transcribed using a reverse-transcription kit (Enzynomics, Daejeon, Korea) following the manufacturer’s instructions. The primers for *18s ribosomal RNA* (*RPS18*)*, SPRY4*, *Aggrecan* (*ACAN*), *SRY-related HMG-box gene 9* (*SOX9*), *Collagen type II* (*COL2*), *RUNX2*, *COL10*, *MMP13, matrix metalloproteinase 1* (*MMP1*)*, matrix metalloproteinase 3* (*MMP3*), and *ADAMTS5* are shown in Supplementary Table 1. qRT-PCR was performed using the StepOnePlus Real-Time PCR System (Applied Biosystems, Warrington, UK) with the Power SYBR Green PCR Master Mix 2x (Applied Biosystems, Warrington, UK).

### Knockdown of *SPRY4* using small interfering RNA (siRNA) in healthy chondrocytes

Chondrocytes were cultured at an initial seeding density of 4 × 10^5^ cells. After 24 h, chondrocytes were transfected with 50 nM of siRNA-SPRY4 (siSPRY4, Bioneer, Daejeon, Korea) using Lipofectamine RNAiMAX Reagent (Invitrogen, CA) and Opti-MEM I Reduced Serum Medium (Gibco, MA) following the standard procedure (*n* = 3). After siRNA treatment, chondrocytes were incubated for 6 h, and then the medium was replaced with fresh low-glucose DMEM. After O/N incubation, chondrocytes were harvested and used in experiments.

### Overexpression of *SPRY4* by lentiviral transduction in DCs

Lentivirus was generated by co-transfection of 2.5 μg of pLenti-SPRY4-mGFP (Origene, MD), 1.25 μg of pMDLg/pRRE (Addgene, MA), 0.625 μg of pRSV-Rev (Addgene, MA), and 0.625 μg of pMD2.G (Addgene, MA) into 293T cells (TaKaRa, Kyoto, Japan) (2 × 10^6^ cells per 100 mm dish) using the ConvoyTM transfection reagent (ACTGene, NJ). After 24 h, the medium was exchanged with a fresh medium. After an additional 24 h incubation at 37 °C, the supernatant containing the lentivirus was collected in a conical tube, and a fresh medium was added to the transfected 293T cells. The lentivirus containing supernatant collected in the conical tube was centrifuged at 1000 rpm for 5 min to remove cellular debris. The supernatant was filtered using a Millex-HV 0.45 μm filter (Merck Millipore, MA) and then ultra-centrifugated at 22,000 rpm for 2 h. The virus pellet was resuspended with a 10 ml fresh growth medium containing 8 ng ml^−1^ hexadimethrine bromide (Sigma Aldrich, MO) and then incubated with chondrocytes for 24 h (*n* = 3).

### Cell viability and proliferation assays

CCK-8 assays (Dojindo, MD) were used to analyze chondrocyte proliferation following the manufacturer’s protocol. Briefly, CCK-8 stock was diluted 1:10 in DMEM low-glucose medium, and 350 µl was added to each well. Treated cells were incubated for 1 h at 37 °C and analyzed with a Cytation 3 Cell Imaging Reader (Biotek, VT) (*n* = 3).

### Western blot analysis

Chondrocytes were washed three times with PBS on ice, lysed with 200 μl RIPA buffer (Sigma Aldrich, MO), and then centrifuged at 13,000 rpm for 20 min. Samples (20 µg) of the total protein extract were then separated by SDS-polyacrylamide gel electrophoresis (SDS-PAGE). Proteins were transferred to nitrocellulose (NC) membrane using a Trans-Blot semi-dry transfer kit (Bio-Rad, CA) and blocked for 1 h with 5% skimmed milk in Tris-buffered saline (TBS) with 0.1% Tween-20 (TBS-T). Blots were incubated O/N with primary antibodies in 5% BSA solution with TBS-T. Membranes were washed 3 times in TBS-T and incubated with horseradish peroxidase-conjugated secondary antibodies (0.1 mg/ml; Jackson Immunoresearch Laboratories) diluted in 5% skimmed milk with TBS-T. Protein bands were analyzed using a Chemi-doc detection system (Bio-Rad, CA).

Primary antibodies for SPRY4 (ab228712, 1:1000 diluted), RUNX2 (ab236639, 1:2000 diluted), ACAN (ab36861, 1:2000 diluted), SOX9 (ab185230, 1:2000 diluted), and ADAMTS5 (ab41037, 1:1000 diluted) were obtained from Abcam (Cambridge, UK). Antibodies for signaling molecules pERK1/2 (4377, 1:1000 diluted), ERK1/2 (9102, 1:2000 diluted), pJNK (4668, 1:1000 diluted), JNK (9258, 1:2000 diluted), pp38 (4631, 1:1000 diluted), and p38 (9212, 1:2000 diluted) were purchased from Cell Signaling Technology (CST) (MA, USA). Antibodies for COL2 (MAB8887, 1:2000 diluted) from Calbiochem (Merck Millipore, MA, USA), antibodies for MMP13 (MAB511-100, 1:1000 diluted) from R&D Systems (MN, USA), and COL10 (C7974-100UL, 1:2000 diluted) from Calbiochem Merck (Merck Millipore, MA, USA). The β-actin (G046, 1:1000 diluted) antibodies were obtained from Applied Biological Materials (Richmond BC, Canada). We ensured that all blots derived from the same experiment and were processed in parallel.

### Flow cytometry

Flow cytometry was used to measure the efficiency of lentiviral transduction into chondrocytes using green fluorescent protein (GFP) expression. Transduced chondrocytes were trypsinized, washed with PBS, and collected in a 1.5 ml tube. For each sample, a suspension of 5 × 10^4^ cells was prepared and used for detection.

### In vivo destabilization of the medial meniscus (DMM) model for osteoarthritis induction

Eight-week-old healthy, male Sprague-Dawley (SD) rats (Orient-bio, Seongnam, Korea) were anesthetized with zoletil (50 mg/kg) and rompun (10 mg/kg). OA was induced by dissecting the medial meniscus to initiate degeneration and collapse of the right knee joint of the hind limb for the OA-induced group (*n* = 4), as described previously^26^. Rats in the control group (*n* = 4) were surgically treated the same without dissecting the medial meniscus. Two rats were housed together in clean, pathogen-free cages with dust-free woodchips as substrate bedding, raised at 55–65% humidity at a controlled temperature of 24 ± 3 °C with a 12 h light/dark cycle, and cared for according to the institutional protocol. At eight weeks post-surgery, all experimental animals were sacrificed, and their right knee joints were collected. Whole staining experiments were performed on the medial tibia cartilage layer of the right knee joint. The experimental protocol was approved by the Institutional Animal Care and Use Committee (IACUC) of CHA University (IACUC180026).

### Statistical analysis

All statistics were analyzed using Prism v8.0 (GraphPad software). A two-tailed unpaired *t*-test with or without Welch’s correction or a Mann Whitney test was used to compare experimental groups following normality (see Supplementary Table 2) and the equal variance test (see Supplementary Table 3). All data were expressed as mean ± standard error of the mean (SEM). The exact *P* values are presented in the figures and summarized in Supplementary Table 4.

### Reporting summary

Further information on research design is available in the Nature Research Reporting Summary linked to this article.

## Supplementary information

Supplementary Information

Reporting Summary

## Data Availability

The data that support the findings of this study are available from the corresponding author upon reasonable request.
