# Supplementary material for: SPRY4 acts as an indicator of osteoarthritis severity and regulates chondrocyte hypertrophy and ECM protease expression
Source: NPJ Regen Med. 2021 Sep 17;6:56. doi: 10.1038/s41536-021-00165-9 (PMC8448831; doi:10.1038/s41536-021-00165-9)
Supplement: Supplementary file 1 — Supplementary Information [file 41536_2021_165_MOESM1_ESM.pdf]

Supplementary Figures

Supplementary Figure 1. *SPRY4* expression in osteoarthritis-induced animal model. Safranin-O staining (upper panel) and SPRY4 IHC staining (lower panel) of rat control cartilage and destabilization of medial meniscus (DMM) model OA-induced cartilage *in vivo*. Scale bar, 100 μm.

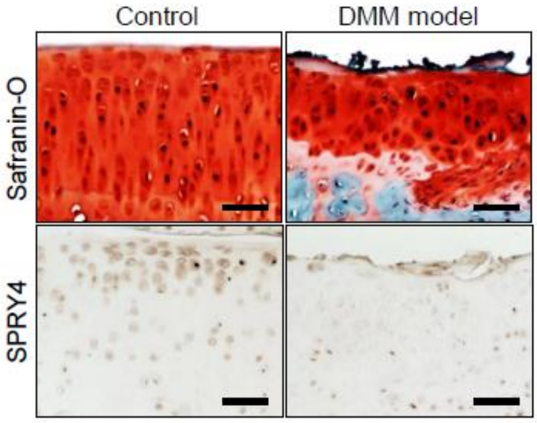

Supplementary Figure 2. Expression of *SPRY4* under OA mimicking conditions in HCs. (a) Chondrogenic marker mRNA expression in untreated and IL-1β-treated HCs (n = 3, unpaired t-test with Welch's correction) (b) ECM protease marker gene expression in untreated and IL-1β-treated HCs (n = 3, unpaired t-test) (c) hypertrophic marker gene expression in untreated and IL-1β-treated HCs (n = 3, unpaired t-test with Welch's correction) (d) *SPRY4* mRNA expression in untreated and IL-1β-treated HC (n = 3, unpaired t-test with Welch's correction) (e) *SPRY4* IF staining of untreated and IL-1β-treated HCs (n = 4, Mann Whitney test) Scale bar, 100 μm. All data represent the mean ± SEM. Abbreviations: IF, immunofluorescence

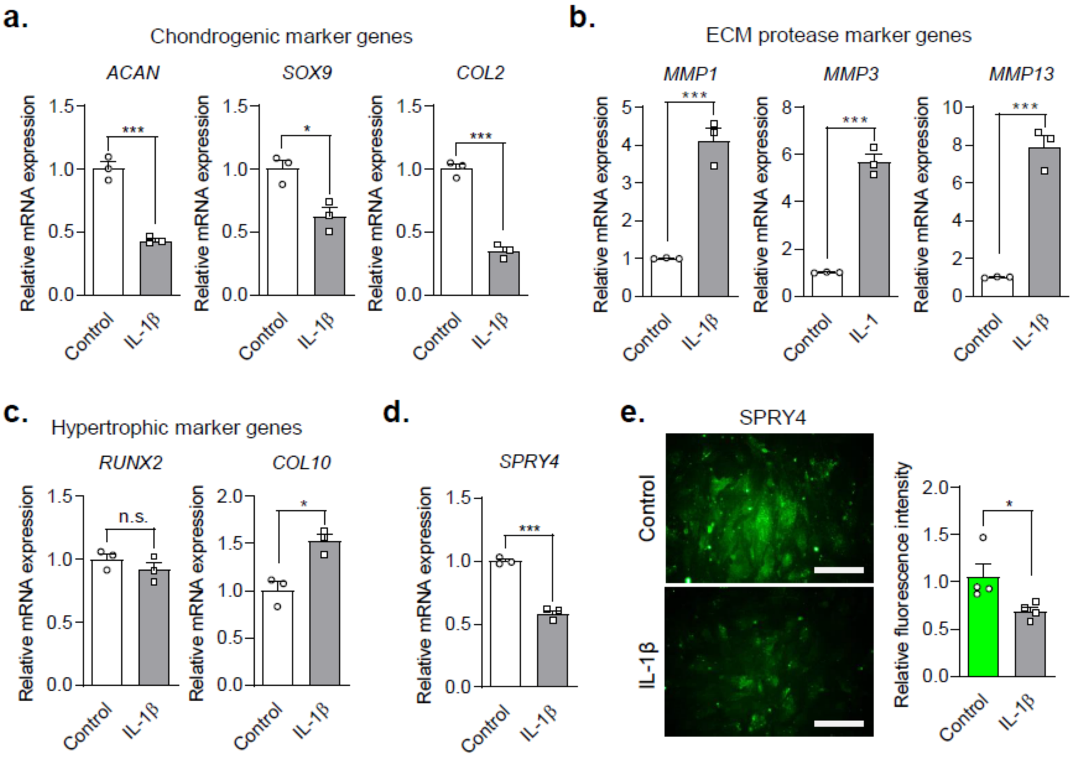

Supplementary Figure 3. Efficiency of *SPRY4* suppression in HCs by siSPRY4. *SPRY4* mRNA expression in siCON-treated and siSPRY4-treated HCs. The data represent the mean ± SEM (n = 3, unpaired t-test with Welch's correction)

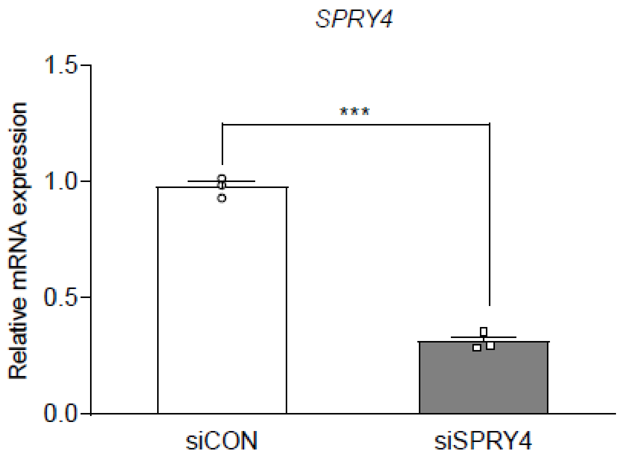

Supplementary Figure 4. The p38 and JNK protein expression after *SPRY4* suppression. pJNK, JNK, pp38, p38, SPRY4, and β-actin protein expression in siCON-treated and siSPRY4-treated HCs.

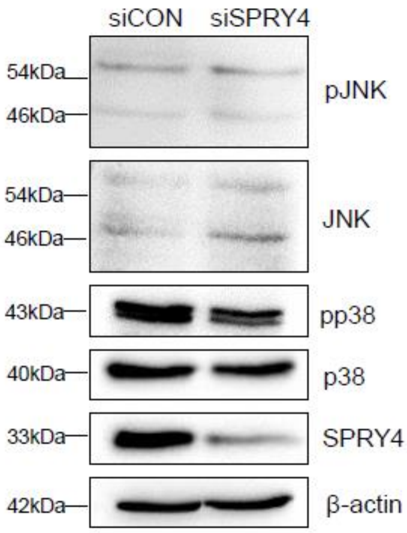

Supplementary Figure 5. Efficiency of *SPRY4* overexpression in DCs by Lenti-*SPRY4*. (a) *SPRY4* mRNA expression in None, Lenti-MOCK, Lenti-GFP, and Lenti-SPRY4-treated DCs. The data represent the mean ± SEM (n = 3, unpaired t-test) (b) Microscopic images (upper panel) and GFP fluorescence images (lower panel) of Lenti-MOCK-treated and Lenti-SPRY4-treated DCs. Scale bars, 200 μm. (c) FACS analysis to determine lentiviral transduction efficiency in Lenti-MOCK-treated and Lenti-SPRY4-treated DCs.

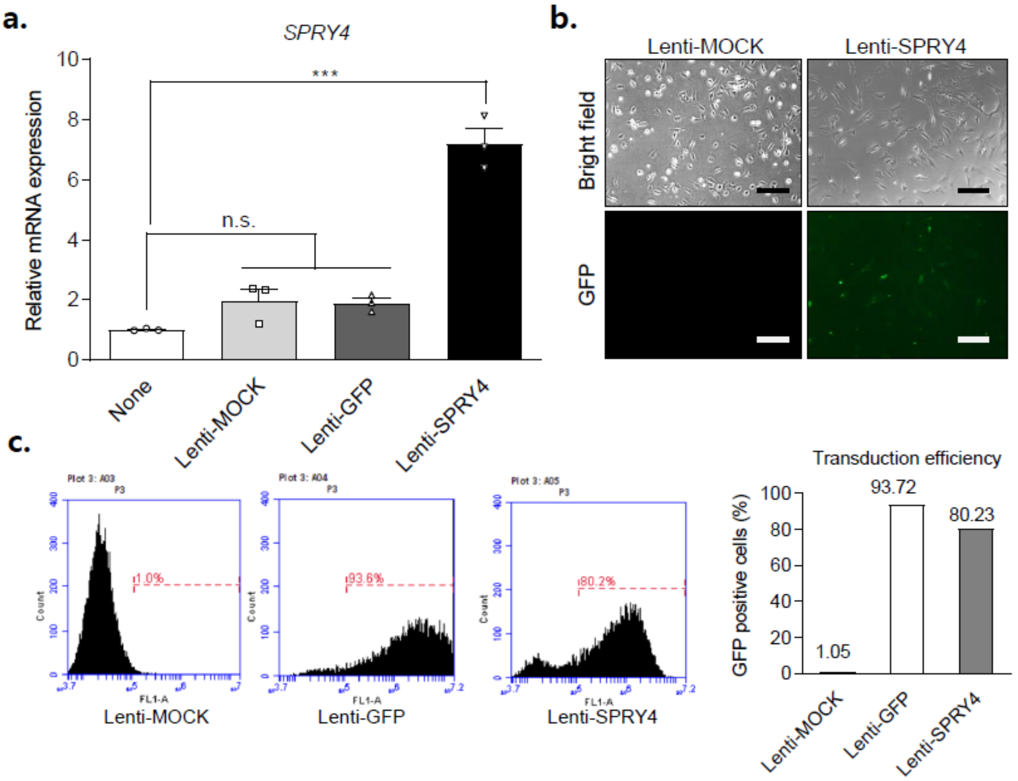

Supplementary Figure 6. Effect of *SPRY4* overexpression on 14-day culture cell viability. CCK-8 cell proliferation assay of control lentivirus (Lenti-CON)-treated and Lenti-SPRY4-treated DCs. The data represent the mean ± SEM (n = 3, unpaired t-test with Welch's correction).

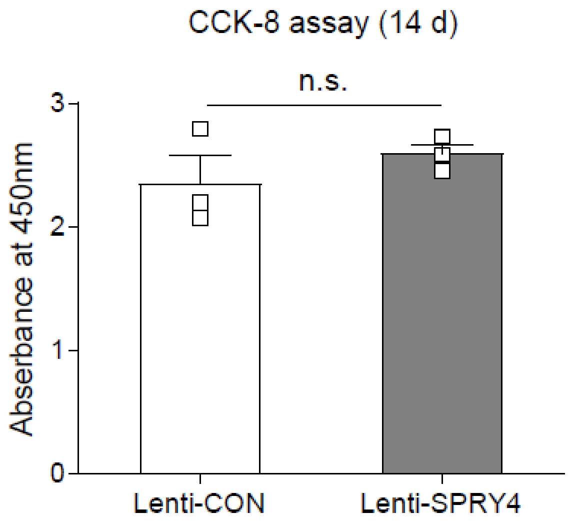

Supplementary Tables

Supplementary Table 1. Nucleotide sequences of primer pairs on qRT-PCR.

qRT-PCR primer sequences list

Gene	Human Primer Sequence	
RPS18	Sense	5′-GTAACCCGTTGAACCCCATT-3′
	Antisense	5′-CCATCCAATCGGTAGTAGCG-3′
SPRY4	Sense	5′-CCTGCAGCTCCTCAAAGG-3′
	Antisense	5′-TGACTGAGTTGGGAGTCAAGG-3′
ACAN	Sense	5′-GCCTGCGCTCCAATGACT-3′
	Antisense	5′-ATGGAACACGATGCCTTTCAC-3′
SOX9	Sense	5′-CCCCAACAGATCGCCTACAG-3′
	Antisense	5′-GAGTTCTGGTCGGTGTAGTC-3′
COL2	Sense	5′-CACGTACACTGCCCTGAAGGA-3′
	Antisense	5′-CGATAACAGTCTTGCCCCACTT-3′
RUNX2	Sense	5′-CAGACCAGCAGCACTCCATA-3′
	Antisense	5′-CAGCGTCAACACCATCATTC-3′
COL10	Sense	5′- ACGCTGAACGATACCAAATG-3′
	Antisense	5′- TGCTATACCTTTACTCTTTATGGTGTA-3′
MMP13	Sense	5′-TCACCAATTCCTGGGAAGTCT-3′
	Antisense	5′- TCAGGAAACCAGGTCTGGAG-3′
MMP1	Sense	5′- GCTAACCTTTGATGCTATAACTACGA-3′
	Antisense	5′- TTTGTGCGCATGTAGAATCTG-3′
MMP3	Sense	5′- CTCACAGACCTGACTCGGTT-3′
	Antisense	5′- AAAGCAGGATCACAGTTGGC-3′
ADAMTS5	Sense	5′- ACAACCAGCTGGGAGATGAC-3′
	Antisense	5′- AAATGTCCGATTTCGTGAGC-3′

Supplementary Table 2. Normality test (Shapiro-Wilk test)

Figure		Group	W	P value	Passed normality test (alpha=0.05)?
Figure 1B	ACAN	HC	0.863	0.2757	Yes
		DC	0.9987	0.9314	Yes
	SOX9	HC	0.9527	0.5811	Yes
		DC	0.9398	0.5264	Yes
	COL2	HC	0.9821	0.7435	Yes
		DC	0.9595	0.613	Yes
	RUNX2	HC	0.9952	0.8671	Yes
		DC	0.9929	0.8384	Yes
	COL10	HC	0.7999	0.1143	Yes
		DC	0.9646	0.6387	Yes
	MMP13	HC	0.9181	0.4458	Yes
		DC	0.7633	0.0296	No
	SPRY4	HC	0.9995	0.9561	Yes
		DC	0.8895	0.3529	Yes
Figure 2A	0 days	siCON	0.9423	0.5367	Yes
		siSPRY4	0.8864	0.3435	Yes
	3 days	siCON	0.8510	0.2431	Yes
		siSPRY4	0.9340	0.5037	Yes
	7 days	siCON	0.8429	0.2216	Yes
		siSPRY4	0.9981	0.9178	Yes
Figure 2B	-	siCON	0.9117	0.478	Yes
		siSPRY4	0.8560	0.2143	Yes
Figure 2C	-	siCON	0.8488	0.1909	Yes
		siSPRY4	0.9949	0.9937	Yes
Figure 2D	-	siCON	0.8918	0.3662	Yes
		siSPRY4	0.9026	0.4243	Yes
Figure 2E	-	siCON	0.8895	0.3806	Yes
		siSPRY4	0.7822	0.0739	Yes
Figure 3A	ACAN	siCON	0.8135	0.1472	Yes
		siSPRY4	0.9757	0.7008	Yes
	SOX9	siCON	0.9944	0.8566	Yes
		siSPRY4	0.9876	0.787	Yes
	COL2	siCON	0.9988	0.9331	Yes
		siSPRY4	0.8932	0.3641	Yes
	RUNX2	siCON	0.9800	0.7291	Yes
		siSPRY4	0.8799	0.324	Yes
	COL10	siCON	0.9694	0.6642	Yes
		siSPRY4	0.9989	0.9352	Yes
	MMP13	siCON	0.8737	0.3061	Yes
		siSPRY4	0.836	0.2038	Yes
	ATAMTS5	siCON	0.8706	0.2972	Yes
		siSPRY4	0.7800	0.0676	Yes
Figure 4A	0 days	Lenti-CON	0.8622	0.2738	Yes
		Lenti-SPRY4	0.9696	0.6653	Yes
	3 days	Lenti-CON	0.9805	0.7327	Yes
		Lenti-SPRY4	0.9984	0.9240	Yes
	7 days	Lenti-CON	0.9991	0.9420	Yes
		Lenti-SPRY4	0.9697	0.6658	Yes
Figure 4B	-	Lenti-CON	0.9577	0.7921	Yes
		Lenti-SPRY4	0.7795	0.0546	Yes
Figure 4C	-	Lenti-CON	0.876	0.2916	Yes
		Lenti-SPRY4	0.8398	0.1643	Yes

Figure 4D	-	Lenti-CON	0.905	0.4379	Yes
		Lenti-SPRY4	0.8792	0.3058	Yes
Figure 4E	-	Lenti-CON	0.8794	0.3067	Yes
		Lenti-SPRY4	0.6906	0.0076	No
Figure 5A	ACAN	Lenti-CON	0.9044	0.3996	Yes
		Lenti-SPRY4	0.7902	0.0913	Yes
	SOX9	Lenti-CON	0.8023	0.1201	Yes
		Lenti-SPRY4	0.9568	0.6001	Yes
	COL2	Lenti-CON	0.8706	0.2974	Yes
		Lenti-SPRY4	0.9108	0.4208	Yes
	RUNX2	Lenti-CON	0.9803	0.7312	Yes
		Lenti-SPRY4	0.8379	0.2085	Yes
	COL10	Lenti-CON	0.8481	0.2353	Yes
		Lenti-SPRY4	0.9952	0.8677	Yes
	MMP13	Lenti-CON	0.9989	0.9358	Yes
		Lenti-SPRY4	0.9988	0.9331	Yes
	ATAMTS5	Lenti-CON	0.85	0.2405	Yes
		Lenti-SPRY4	0.7837	0.0762	Yes
Figure S1A	ACAN	Control	0.9998	0.9749	Yes
		IL-1β	0.9267	0.4763	Yes
	SOX9	Control	0.8705	0.297	Yes
		IL-1β	0.9956	0.8738	Yes
	COL2	Control	0.8841	0.3365	Yes
		IL-1β	0.9801	0.7293	Yes
Figure S1B	MMP1	Control	0.7872	0.0842	Yes
		IL-1β	0.9014	0.3898	Yes
	MMP3	Control	0.9904	0.8128	Yes
		IL-1β	0.9804	0.7317	Yes
	MMP13	Control	0.9546	0.59	Yes
		IL-1β	0.8611	0.2707	Yes
Figure S1C	RUNX2	Control	0.8927	0.3625	Yes
		IL-1β	0.9975	0.9041	Yes
	COL10	Control	0.8493	0.2387	Yes
		IL-1β	0.9264	0.4751	Yes
Figure S1D	SPRY4	Control	0.9372	0.5164	Yes
		IL-1β	0.8347	0.2004	Yes
Figure S1E	-	Control	0.7346	0.0274	No
		IL-1β	0.9898	0.9565	Yes
Figure S2	-	siCON	0.9727	0.6829	Yes
		siSPRY4	0.8741	0.3073	Yes
Figure S3A	-	None	0.8452	0.2278	Yes
		Lenti-MOCK	0.7799	0.0673	Yes
		Lenti-GFP	0.9986	0.9296	Yes
		Lenti-SPRY4	0.9881	0.7913	Yes

Supplementary Table 3. Equal variance test (F test)

Figure		F, DFn, Dfd	P value	Yes or No
Figure 1B	ACAN	73.11, 2, 2	0.0270	Yes
	SOX9	50.31, 2, 2	0.0390	Yes
	COL2	197.2, 2, 2	0.0101	Yes
	RUNX2	3.874, 2, 2	0.4104	No
	COL10	25.14, 2, 2	0.0765	No
	MMP13	1259, 2, 2	0.0016	Yes
	SPRY4	1.538, 2, 2	0.7879	No
Figure 2A	0 days	13.56, 2, 2	0.1374	No
	3 days	7.087, 2, 2	0.2473	No
	7 days	30.68, 2, 2	0.0631	No
Figure 2B	-	13.35, 4, 4	0.0278	Yes
Figure 2C	-	5.564, 4, 4	0.1251	No
Figure 2D	-	3.588, 4, 4	0.2437	No
Figure 2E	-	25.93, 3, 3	0.0240	Yes
Figure 3A	ACAN	4.728, 2, 2	0.3492	No
	SOX9	9.031, 2, 2	0.1994	No
	COL2	12.38, 2, 2	0.1495	No
	RUNX2	27.35, 2, 2	0.0705	No
	COL10	1.433, 2, 2	0.8221	No
	MMP13	29.25, 2, 2	0.0661	No
	ADAMTS5	10.76, 2, 2	0.1700	No
Figure 4A	0 days	1.074, 2, 2	0.9641	No
	3 days	1.784, 2, 2	0.7184	No
	7 days	11.74, 2, 2	0.1570	No
Figure 4B	-	3.346, 4, 4	0.2689	No
Figure 4C	-	9.504, 4, 4	0.0509	No
Figure 4D	-	18.84, 4, 4	0.0147	Yes
Figure 4E	-	2.420, 4, 4	0.4130	No
Figure 5A	ACAN	84.00, 2, 2	0.0235	Yes
	SOX9	6.378, 2, 2	0.2711	No
	COL2	4.740, 2, 2	0.3484	No
	RUNX2	3.125, 2, 2	0.4848	No
	COL10	1.560, 2, 2	0.7813	No
	MMP13	266.4, 2, 2	0.0075	Yes
	ADAMTS5	1.829, 2, 2	0.7069	No
Figure S1A	ACAN	12.48, 2, 2	0.1483	No
	SOX9	1.066, 2, 2	0.9678	No
	COL2	1.256, 2, 2	0.8865	No
Figure S1B	MMP1	1315, 2, 2	0.0015	Yes
	MMP3	471.0, 2, 2	0.0042	Yes
	MMP13	807.2, 2, 2	0.0025	Yes
Figure S1C	RUNX2	1.666, 2, 2	0.7501	No
	COL10	1.418, 2, 2	0.8270	No
Figure S1D	-	3.003, 2, 2	0.4997	No
Figure S1E	-	10.35, 3, 3	0.0865	No
Figure S2	-	1.268, 2, 2	0.8819	No
Figure S3	None vs. Lenti-MOCK	324.6, 2, 2	0.0061	Yes
	None vs. Lenti-GFP	57.24, 2, 2	0.0343	Yes
	None vs. Lenti-GFP	592.4, 2, 2	0.0034	Yes

Supplementary Table 4. Exact *P* values and group differences of interest with 95% CIs

Figure	Comparison	P value	T, df	95% CI	R squared	test
Figure 1B	ACAN: HC vs. DC	<0.0001	54.34, 4	-0.8694 to -0.7849	0.9986	Unpaired t test
	SOX9: HC vs. DC	<0.0001	33.66, 4	-0.8279 to -0.7018	0.9965	Unpaired t test
	COL2: HC vs. DC	<0.0001	131.0, 4	-0.9813 to -0.9405	0.9998	Unpaired t test
	RUNX2: HC vs. DC	0.0006	15.51, 2.968	2.238 to 3.214	0.9836	Unpaired t test with Welch's correction
	COL10: HC vs. DC	0.0552	3.814, 2.159	-0.8861 to -0.1395	0.7843	Unpaired t test with Welch's correction
	MMP13: HC vs. DC	0.100	n/a	n/a	n/a	Mann Whitney test
	SPRY4: HC vs. DC	0.0013	8.447, 3.828	-0.4415 to -0.2231	0.9469	Unpaired t test with Welch's correction
Figure 2A	0 days: siCON vs. siSPRY4	0.0652	3.347, 2.293	-0.007452 to 0.1138	0.8300	Unpaired t test with Welch's correction
	3 days: siCON vs. siSPRY4	0.0014	15.30, 2.553	-0.9128 to -0.5712	0.9892	Unpaired t test with Welch's correction
	7 days: siCON vs. siSPRY4	0.0043	13.39, 2.130	-0.9505 to -0.5081	0.9883	Unpaired t test with Welch's correction
Figure 2B	siCON vs. siSPRY4	0.0061	3.696, 8	0.6421 to 2.773	0.6306	Unpaired t test
Figure 2C	siCON vs. siSPRY4	0.0031	5.075, 5.393	0.4967 to 1.324	0.763	Unpaired t test with Welch's correction
Figure 2D	siCON vs. siSPRY4	0.0001	8.934, 6.069	0.4486 to 0.7607	0.9089	Unpaired t test with Welch's correction
Figure 2E	siCON vs. siSPRY4	<0.0001	11.77, 6	11.64 to 17.75	0.9585	Unpaired t test
Figure 3A	ACAN: siCON vs. siSPRY4	<0.0001	36.16, 2.810	-0.7646 to -0.6556	0.997	Unpaired t test with Welch's correction
	SOX9: siCON vs. siSPRY4	0.0002	37.50, 2.438	-0.7617 to -0.6567	0.9972	Unpaired t test with Welch's correction
	COL2: siCON vs. siSPRY4	0.0003	37.47, 2.321	-1.061 to -0.9149	0.9972	Unpaired t test with Welch's correction
	RUNX2: siCON vs. siSPRY4	0.0002	50.51, 2.146	0.9366 to 1.045	0.9984	Unpaired t test with Welch's correction
	COL10: siCON vs. siSPRY4	0.0003	12.27, 3.877	0.4520 to 0.7165	0.9741	Unpaired t test with Welch's correction
	MMP13: siCON vs. siSPRY4	0.0022	18.43, 2.137	5.373 to 7.279	0.9884	Unpaired t test with Welch's correction

	ADAMTS5: siCON vs. siSPRY4	0.0009	21.06, 2.368	0.2870 to 0.3742	0.9911	Unpaired t test with Welch's correction
Figure 4A	0 days: Lenti-CON vs. Lenti-SPRY4	0.4980	0.7445, 3.995	-0.04734 to 0.08201	0.1218	Unpaired t test with Welch's correction
	3 days: Lenti-CON vs. Lenti-SPRY4	0.3501	1.068, 3.706	-0.02469 to 0.05402	0.2353	Unpaired t test with Welch's correction
	7 days: Lenti-CON vs. Lenti-SPRY4	0.9450	0.07665, 2.338	-0.4002 to 0.4169	0.002506	Unpaired t test with Welch's correction
Figure 4B	Lenti-CON vs. Lenti-SPRY4	0.0188	3.156, 6.195	-1.012 to -0.1574	0.5546	Unpaired t test with Welch's correction
Figure 4C	Lenti-CON vs. Lenti-SPRY4	0.0471	2.648, 4.833	-0.8770 to -0.06059	0.4671	Unpaired t test with Welch's correction
Figure 4D	Lenti-CON vs. Lenti-SPRY4	0.0467	2.350, 8	-0.9871 to -0.009298	0.4084	Unpaired t test
Figure 4E	Lenti-CON vs. Lenti-SPRY4	0.0556	n/a	n/a	n/a	Mann Whitney test
Figure 5A	ACAN: Lenti-CON vs. Lenti-SPRY4	0.0089	4.763, 4	0.06476 to 0.2458	0.8501	Unpaired t test
	SOX9: Lenti-CON vs. Lenti-SPRY4	0.0230	4.835, 2.612	-0.3080 to -0.08330	0.8539	Unpaired t test with Welch's correction
	COL2: Lenti-CON vs. Lenti-SPRY4	0.2302	1.527, 2.808	-0.9477 to 0.2751	0.3683	Unpaired t test with Welch's correction
	RUNX2: Lenti-CON vs. Lenti-SPRY4	0.0025	8.775, 3.161	-0.3841 to -0.1995	0.9506	Unpaired t test with Welch's correction
	COL10: Lenti-CON vs. Lenti-SPRY4	0.0016	8.007, 3.817	-0.5111 to -0.2479	0.9413	Unpaired t test with Welch's correction
	MMP13: Lenti-CON vs. Lenti-SPRY4	<0.0001	131.5, 4	-0.7209 to -0.6911	0.9998	Unpaired t test
	ADAMTS5: Lenti-CON vs. Lenti-SPRY4	0.0003	12.83, 3.684	-0.5665 to -0.3650	0.9763	Unpaired t test with Welch's correction
Figure S1A	ACAN: Control vs. IL-1β	0.0054	10.34, 2.318	-0.7261 to -0.4188	0.964	Unpaired t test with Welch's correction
	SOX9: Control vs. IL-1β	0.0148	4.104, 3.996	-0.6353 to -0.1226	0.8081	Unpaired t test with Welch's correction
	COL2: Control vs. IL-1β	0.0002	13.40, 3.949	-0.7884 to -0.5177	0.9782	Unpaired t test with Welch's correction
Figure S1B	MMP1: Control vs. IL-1β	0.0008	9.018, 4	2.155 to 4.072	0.9531	Unpaired t test
	MMP3: Control vs. IL-1β	0.0001	14.28, 4	3.773 to 5.594	0.9808	Unpaired t test
	MMP13: Control vs. IL-1β	0.0004	11.16, 4	5.166 to 8.586	0.9689	Unpaired t test

Figure S1C	RUNX2: Control vs. IL-1β	0.3212	1.141, 3.765	-0.2853 to 0.1191	0.2456	Unpaired t test with Welch's correction
	COL10: Control vs. IL-1β	0.0114	4.526, 3.884	0.2015 to 0.8412	0.8366	Unpaired t test with Welch's correction
Figure S1D	Control vs. IL-1β	0.0006	13.52, 3.199	-0.5031 to -0.3316	0.9786	Unpaired t test with Welch's correction
Figure S1E	Control vs. IL-1β	0.0286	n/a	n/a	n/a	Mann Whitney test
Figure S2	siCON vs. siSPRY4	<0.0001	19.97, 3.945	-0.7565 to -0.5718	0.9901	Unpaired t test with Welch's correction
Figure S3	None vs. Lenti-MOCK	0.0626	2.561, 4	-0.08128 to 2.017	0.6212	Unpaired t test
	None vs. Lenti-GFP	0.0052	5.546, 4	0.4426 to 1.330	0.8849	Unpaired t test
	None vs. Lenti-SPRY4	0.0003	12.14, 4	4.774 to 7.606	0.9736	Unpaired t test

The uncropped western blots

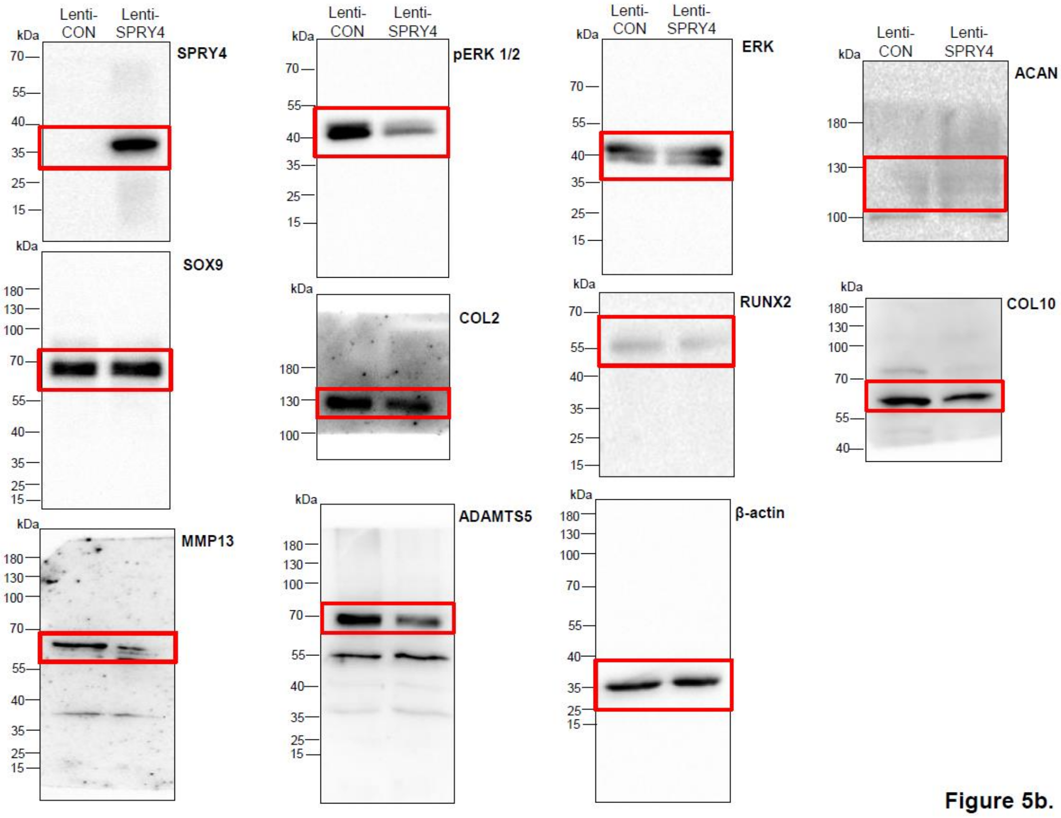

Figure 5b.

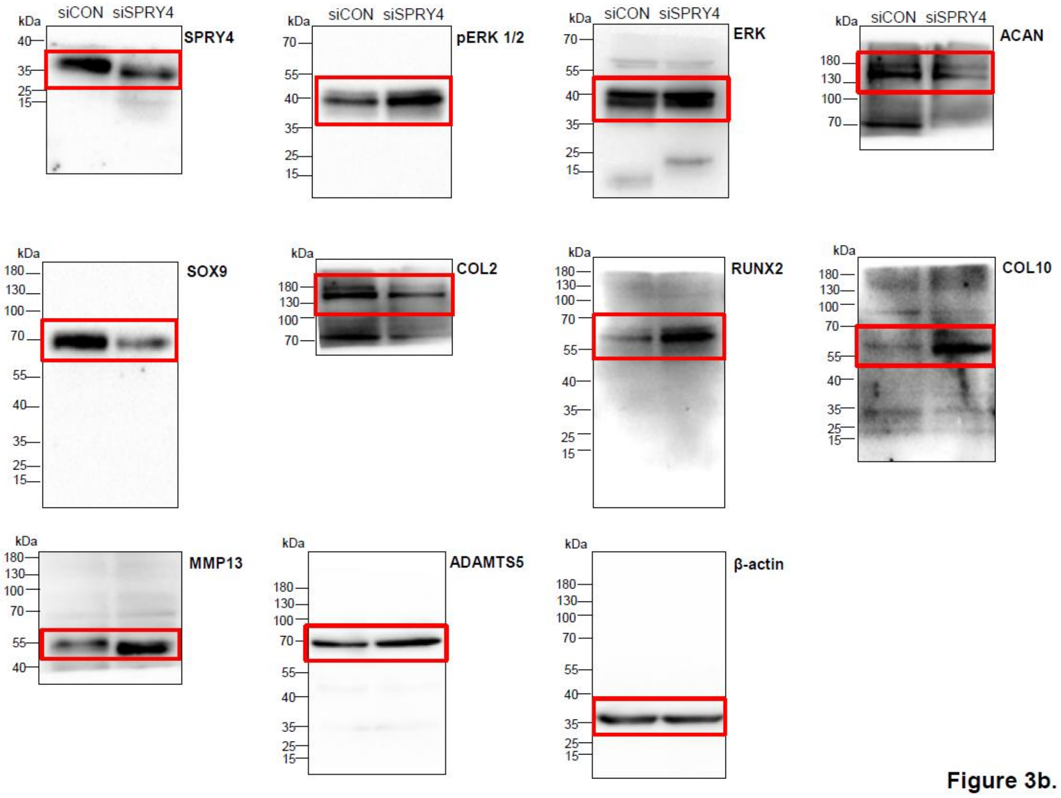

Figure 3b.

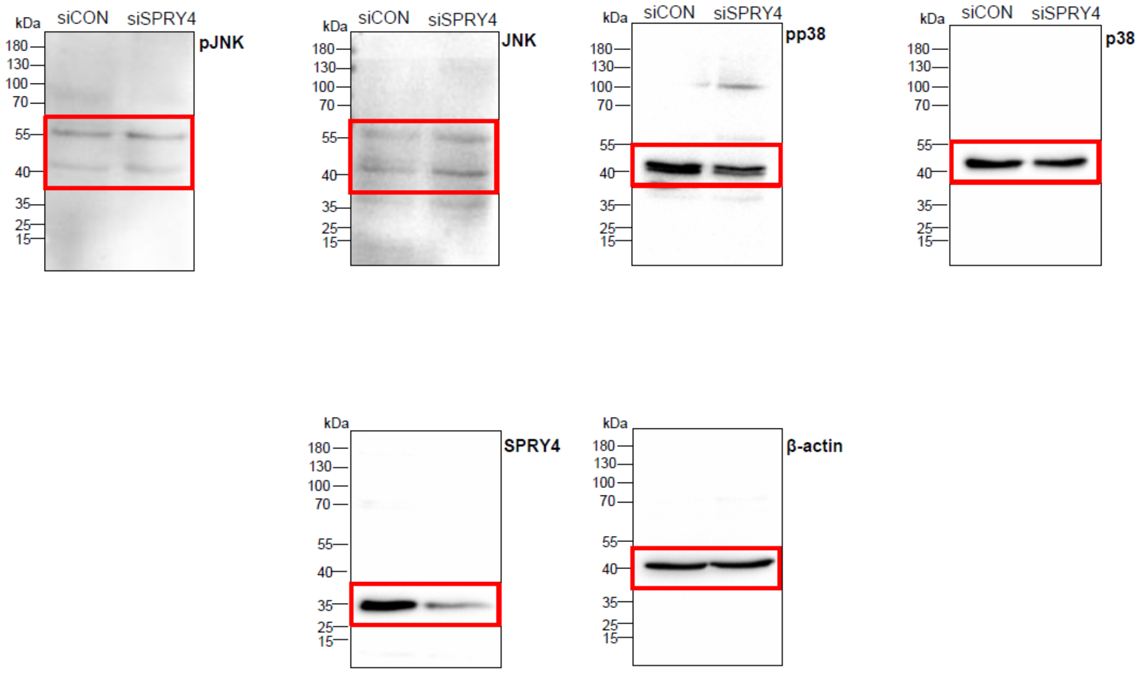

Supplementary figure 4.
