# Supplementary material for: SPRY4 acts as an indicator of osteoarthritis severity and regulates chondrocyte hypertrophy and ECM protease expression
Source: NPJ Regen Med. 2021 Sep 17;6:56. doi: 10.1038/s41536-021-00165-9 (PMC8448831; doi:10.1038/s41536-021-00165-9)
Supplement: Supplementary file 2 — Reporting Summary [file 41536_2021_165_MOESM2_ESM.pdf]

nature portfolio

Corresponding author(s): Soo-Hong Lee

Last updated by author(s): Aug 2, 2021

Reporting Summary

Nature Portfolio wishes to improve the reproducibility of the work that we publish. This form provides structure for consistency and transparency in reporting. For further information on Nature Portfolio policies, see our Editorial Policies and the Editorial Policy Checklist.

Statistics

For all statistical analyses, confirm that the following items are present in the figure legend, table legend, main text, or Methods section.

n/a	Confirmed	
☐	☒	The exact sample size (n) for each experimental group/condition, given as a discrete number and unit of measurement
☒	☐	A statement on whether measurements were taken from distinct samples or whether the same sample was measured repeatedly
☐	☒	The statistical test(s) used AND whether they are one- or two-sided<br>*Only common tests should be described solely by name; describe more complex techniques in the Methods section.*
☒	☐	A description of all covariates tested
☐	☒	A description of any assumptions or corrections, such as tests of normality and adjustment for multiple comparisons
☐	☒	A full description of the statistical parameters including central tendency (e.g. means) or other basic estimates (e.g. regression coefficient) AND variation (e.g. standard deviation) or associated estimates of uncertainty (e.g. confidence intervals)
☐	☒	For null hypothesis testing, the test statistic (e.g. F, t, r) with confidence intervals, effect sizes, degrees of freedom and P value noted<br>*Give P values as exact values whenever suitable.*
☒	☐	For Bayesian analysis, information on the choice of priors and Markov chain Monte Carlo settings
☒	☐	For hierarchical and complex designs, identification of the appropriate level for tests and full reporting of outcomes
☒	☐	Estimates of effect sizes (e.g. Cohen's d, Pearson's r), indicating how they were calculated

Our web collection on statistics for biologists contains articles on many of the points above.

Software and code

Policy information about availability of computer code

Data collection: *Provide a description of all commercial, open source and custom code used to collect the data in this study, specifying the version used OR state that no software was used.*

Data analysis: *Provide a description of all commercial, open source and custom code used to analyse the data in this study, specifying the version used OR state that no software was used.*

For manuscripts utilizing custom algorithms or software that are central to the research but not yet described in published literature, software must be made available to editors and reviewers. We strongly encourage code deposition in a community repository (e.g. GitHub). See the Nature Portfolio guidelines for submitting code & software for further information.

Data

Policy information about availability of data

All manuscripts must include a data availability statement. This statement should provide the following information, where applicable:

- Accession codes, unique identifiers, or web links for publicly available datasets
- A description of any restrictions on data availability
- For clinical datasets or third party data, please ensure that the statement adheres to our policy

All data that performed or analyzed of this study are included in this article with its Supplementary figures and tables.
The data that support the findings of this study are available from the corresponding author upon reasonable request.

Field-specific reporting

Please select the one below that is the best fit for your research. If you are not sure, read the appropriate sections before making your selection.

☒ Life sciences ☐ Behavioural & social sciences ☐ Ecological, evolutionary & environmental sciences

For a reference copy of the document with all sections, see nature.com/documents/nr-reporting-summary-flat.pdf

Life sciences study design

All studies must disclose on these points even when the disclosure is negative.

Sample size	All experiments were conducted with 90% power and a significance of 0.05.
Data exclusions	NA
Replication	In vitro data were performed by three independent experiments.
Randomization	Rats and chondrocytes were randomly allocated into control group and treatment group.
Blinding	Experiments and assessments were blinded.

Reporting for specific materials, systems and methods

We require information from authors about some types of materials, experimental systems and methods used in many studies. Here, indicate whether each material, system or method listed is relevant to your study. If you are not sure if a list item applies to your research, read the appropriate section before selecting a response.

Materials & experimental systems

n/a	Involved in the study	
☐	☒	Antibodies
☐	☒	Eukaryotic cell lines
☒	☐	Palaeontology and archaeology
☐	☒	Animals and other organisms
☒	☐	Human research participants
☒	☐	Clinical data
☒	☐	Dual use research of concern

Methods

n/a	Involved in the study	
☒	☐	ChIP-seq
☒	☐	Flow cytometry
☒	☐	MRI-based neuroimaging

Antibodies

Antibodies used	Rabbit Anti-SPRY4 antibody (100 µg at 0.9 - 1 mg/ml, abcam, hanam, korea), Rabbit Anti-RUNX2 antibody (100 µl at 0.412 - 0.448 mg/ml, abcam, hanam, korea), Rabbit Anti-Aggrecan antibody (100 µg at 0.9 - 1 mg/ml, abcam, hanam, korea), Rabbit Anti-SOX9 antibody (100 µl at 1.236 mg/ml, abcam, hanam, korea), Rabbit Anti-ADAMTS5 antibody (100 µg at 1 mg/ml, abcam, hanam, korea), Rabbit Anti-p-p44/42(ERK 1/2) antibody (200 µl, CST, MA, US), Rabbit Anti-p44/42(ERK 1/2) antibody (200 µl, CST, MA, US), Rabbit-Anti-p-SAPK/JNK (Thr183/Tyr185) antibody (100 µl, CST, MA, US), Rabbit-Anti-JNK antibody (200 µl, CST, MA, US), Rabbit-Anti-p-p38 MAPK antibody (200 µl, CST, MA, US), Rabbit-Anti-p38 MAPK antibody (200 µl, CST, MA, US), Mouse-Anti-Collagen Type II antibody (100 µl, Merck Millipore, MA, US), Mouse-Anti-Collagen MMP13 antibody (100 µg at 0.5 mg/ml, R&D Systems, MN, USA), Mouse-Anti-Collagen type X antibody (100 µl ,Merck Millipore, MA, US), Mouse-Anti-beta-actin antibody (1.0 mg/ml, Richmond BC, canada)
Validation	Each antibody were validated by manufacturers protocols.

Eukaryotic cell lines

Policy information about cell lines

Cell line source(s)	Human chondrocytes obtained from knee joint cartilage of human OA patients
Authentication	Human chondrocytes obtained from knee joint cartilage of human OA patients
Mycoplasma contamination	Human chondrocytes were tested negative to mycoplasma contamination via MycoAlertTM Mycoplasma Detection Kit Lonza, Basel, Switzerland.
Commonly misidentified lines (See ICLAC register)	*Name any commonly misidentified cell lines used in the study and provide a rationale for their use.*

Animals and other organisms

Policy information about studies involving animals; ARRIVE guidelines recommended for reporting animal research

Laboratory animals	8-week-old male Sprague-Dawley (SD) rats (Orient-bio, Seongnam, Korea).
Wild animals	*Provide details on animals observed in or captured in the field; report species, sex and age where possible. Describe how animals were caught and transported and what happened to captive animals after the study (if killed, explain why and describe method; if released, say where and when) OR state that the study did not involve wild animals.*
Field-collected samples	*For laboratory work with field-collected samples, describe all relevant parameters such as housing, maintenance, temperature, photoperiod and end-of-experiment protocol OR state that the study did not involve samples collected from the field.*
Ethics oversight	This study was approved by the Institutional Animal Care and Use Committee (IACUC) of CHA University (IACUC180026).

Note that full information on the approval of the study protocol must also be provided in the manuscript.
